# Low-frequency lattice vibrations from atomic displacement parameters of α-FOX-7, a high energy density material

**DOI:** 10.1107/S2052520622002700

**Published:** 2022-05-11

**Authors:** Thammarat Aree, Charles J. McMonagle, Adam A. L. Michalchuk, Dmitry Chernyshov

**Affiliations:** a Department of Chemistry, Faculty of Science, Chulalongkorn University, Bangkok, 10330, Thailand; b Swiss-Norwegian Beam Lines (SNBL) at ESRF, Grenoble, France; c Federal Institute for Materials Research and Testing (BAM), Berlin, Germany

**Keywords:** α-FOX-7, anisotropic atomic displacement parameters, ADPs, normal mode analysis, crystal dynamics

## Abstract

Highly anharmonic thermal vibrations may serve as a source of structural instabilities resulting in phase transitions, chemical reactions and even the mechanical disintegration of a material. *Ab initio* calculations model thermal motion within a harmonic or sometimes quasi-harmonic approximation and must be com­plimented by experimental data on tem­per­ature-dependent vibrational frequencies. Here multi-tem­per­ature atomic displacement parameters (ADPs), derived from a single-crystal synchrotron diffraction experiment, are used to characterize low-frequency lattice vibrations in the α-FOX-7 layered structure. It is shown that despite the limited quality of the data, the extracted frequencies are reasonably close to those derived from inelastic scattering, Raman measurements and density functional theory (DFT) calculations. Vibrational anharmonicity is parameterized by the Grüneisen parameters, which are found to be very different for in-layer and out-of-layer vibrations.

## Abstract 2

The normal mode analysis of variable-tem­per­ature anisotropic atomic displacement parameters (ADPs) of the α-phase of 1,1-di­amino-2,2-di­nitro­ethyl­ene (DADNE or FOX-7) is reported.

## 1. Introduction

FOX-7 (DADNE, C_2_H_4_N_4_O_4_) (Latypov *et al.*, 1998 ▸; Trzciński & Belaada, 2016 ▸) is a high energy density material (HEDM) that shows a set of phase transformations as a function of tem­per­ature and/or pressure (Bu *et al.*, 2020 ▸). The structural and mechanical stability of different polymorphs are defined, among other factors, by their thermal mol­ecular vibrations. The structures of all reported crystalline forms of α-, β- and γ-FOX-7 are built from wave-shaped layers of FOX-7 mol­ecules linked by hydrogen bonding and the stacked layers are weakly bound by van der Waals inter­actions (Crawford *et al.*, 2007 ▸).

The mechanisms for the impact-induced initiation of HEDMs has been debated for many decades. Understanding the initiation process is essential for designing safe and well-performing materials suitable for use across military and civilian applications. Building on early numerical models (Tokmakoff *et al.*, 1993 ▸), recent theoretical developments have suggested that initiation is strongly correlated to the dynamic behaviour of the material (Michalchuk *et al.*, 2019 ▸, 2021*a* ▸). When struck by a mechanical force, the energy is inserted into the lattice vibrations, up-converting through phonon–phonon collisions until the mol­ecules are vibrationally excited. If sufficiently excited, covalent-bond rupture occurs, leading to the primary initiation event. These so-called ‘phonon up-pumping’ models have proved to be very promising across a wide range of HEDMs, though their further development requires detailed investigations into the structural dynamics of these materials. In connection with this, FOX-7 is of particular inter­est, owing to its polymorphic behaviour and layered crystal packing, the latter widely believed to indicate insensitivity to impact initiation (Ma *et al.*, 2014 ▸). Recent studies have suggested that FOX-7 may undergo a polymorphic transformation in response to mechanical impact (Michalchuk *et al.*, 2021*b* ▸), although the influence of such transformations on material performance is not known. Within the framework of phonon up-pumping, a thorough understanding of FOX-7 dynamics is needed before its com­plex initiation behaviour can be further elucidated.

The use of neutron or X-ray inelastic scattering for studying vibrational properties is rather limited for FOX-7 due to the low mol­ecular symmetry and high lattice anharmonicity, and hence relatively large crystals of FOX-7 are required. This is why only phonon density of states measured with neutron inelastic scattering from powders have been reported so far (Hunter *et al.*, 2015 ▸; Michalchuk *et al.*, 2019 ▸). Moreover, the presence of H atoms represents a problem for coherent neutron scattering experiments. Raman and IR spectroscopies provide information on the vibrational frequencies at the Γ-point of the Brillouin zone and are typically restricted to the analysis of wavenumbers > 50–100 cm^−1^; data on low-fre­quency vibrations and dispersion are very limited. The majority of spectroscopic techniques are focused on vibrational frequencies (eigenvalues of the dynamical matrices), while phonon eigenvectors are characterized experimentally for very few structurally simple materials (Strauch & Dorner, 1986 ▸; Pawley *et al.*, 1980 ▸). Here we have applied a concurrent analysis of the variable-tem­per­ature ADPs routinely derived from diffraction experiment (Bürgi & Capelli, 2000 ▸) to investigate the lattice vibration properties of the monoclinic α-phase of FOX-7. This approach considers eigenvectors of dynamical matrices, which are encoded in the shape of displacement ellipsoids – smearing of atoms in the diffraction experiment. The phonon modes are modelled with line spectra as Einstein oscillators. For a mol­ecular material, thermal vibrations are split on the rigid unit modes (RUMs) – displacements of a mol­ecule as a whole (translations and librations), and deformations associated with optic phonons that normally have higher frequencies. While this approximation is appropriate for all mol­ecular solids, particularly where ‘soft’ mol­ecular modes exist, such as –NO_2_ wags, it holds well for crystalline FOX-7 (Michalchuk *et al.*, 2021*a* ▸,*b* ▸).

The monoclinic α-phase of FOX-7 is stable below 380 K. Thus far, only one set of single-crystal X-ray (Mo *K*α) diffraction data containing five tem­per­ature points in the range 200–293 K at ambient pressure, to *d*_min_ of 0.76 Å, is available (Evers *et al.*, 2006 ▸). Additional powder data at 403 and 423 K have also been collected, indicating the first-order α→β phase transition (monoclinic *P*2_1_/*n* → ortho­rhom­bic *P*2_1_2_1_2_1_) at 389 K (Evers *et al.*, 2006 ▸). The out-of-plane displacements of four O atoms in two –NO_2_ groups are clearly observed in both phases of FOX-7 in the range 200–423 K (Evers *et al.*, 2006 ▸). More attention has been paid to FOX-7 under high pressure–tem­per­ature conditions, combining experiment and simulation to study its phase transitions, structural changes and vibrational behaviour (Peiris *et al.*, 2004 ▸; Hu *et al.*, 2006 ▸; Bishop *et al.*, 2012 ▸; Dreger *et al.*, 2013 ▸, 2014 ▸; Appalakondaiah *et al.*, 2014 ▸; Hunter *et al.*, 2015 ▸). Here we com­plement the available information on thermal vibrations with low-energy frequencies (librations and translations) and their anharmonic behaviour parameterized with Grün­eisen parameters. We also include in the analysis some of the vibrations associated with deformation of the FOX-7 mol­ecule, providing results that are reasonably close to the reported values for low-frequency optic phonons at the Γ-point of the Brillouin zone, as expected for the phonon modes with low dispersion.

The collection of diffraction data with synchrotron light can be easily done with very small crystals; high intensity and fast detectors reduce the data collection time to tens of seconds and the data can be collected with very fine tem­per­ature sampling. However, the data quality frequently suffers from a nonhomogeneous and/or unstable beam, an irregular shape of the crystal or inadequately characterized attenuation of the incoming and scattered beams with crystal mounts; all these effects are believed to be minimized with empirical absorption and scaling corrections. ADPs are the most sensitive parameters and may therefore contain an additional contribution not related to thermal smearing but rather linked to the data and data processing, as demonstrated by the simultaneous analysis of multi-tem­per­ature ADPs of the three glycine polymorphs (Aree & Bürgi, 2012 ▸; Aree *et al.*, 2013 ▸, 2014 ▸). Here we show that those contributions, being tem­per­ature independent, do not distort information on the low-energy thermal vibrations. To improve information concerning the dynamics of H atoms and general quality of ADPs, we have applied a nonspherical refinement of X-ray diffraction data developed by Kleemiss *et al.* (2021 ▸). Altogether, the data col­lection, data processing and structure refinement applied here show that a vibrational analysis similar to that presented here might become a relatively simple-to-use tool that offers unique information and can be easily implemented for single-crystal diffraction experiments at synchrotron beamlines.

## 2. Experimental

### 2.1. Materials

For the synthesis of 1,1-di­amino-2,2-di­nitro­ethene (DADNE or FOX-7), 2-methyl­pyrimidine-4,6-dione (3.0 g, 0.025 mol) was dissolved in H_2_SO_4_ (95%, 25 ml) at tem­per­atures < 303 K. HNO_3_ (99%, 10 ml) was added dropwise, ensuring that the tem­per­ature remained below 293 K during addition. The sample was stirred on ice for 3 h. The resulting material was rinsed with H_2_SO_4_ (95%) and dissolved in deionized water. The precipitated product was filtered off and dried. Single crystals of FOX-7 were grown by slow evaporation from dimethyl sulfoxide (DMSO).

### 2.2. Multi-tem­per­ature single-crystal X-ray diffraction

#### 2.2.1. Synchrotron diffraction experiment

A single crystal of α-FOX-7 (0.2 × 0.1 × 0.1 mm) was selected and mounted on the tip of a glass fibre with the minimum of high-temper­ature ep­oxy. Variable-tem­per­ature single-crystal diffraction data were collected at the Swiss–Norwegian Beamline BM01 (ESRF, Grenoble) (Dyadkin *et al.*, 2016 ▸) upon heating from 80 to 360 K, with tem­per­ature control by an Oxford Cryosystems Cryostream cooler (Cosier & Glazer, 1986 ▸). Fifty eight temper­ature data points with a 4–8 K increment were collected. For each tem­per­ature, a full data collection was carried out at a wavelength of 0.62379 Å (19.876 keV) with a single ω-scan. Moreover, multi-tem­per­ature data were continuously col­lected to 474 K and the data between 274 and 474 K were used for an investigation of the thermal expansion and phase transitions of the energetic material FOX-7 (McMonagle *et al.*, 2022 ▸). This work shared data between 80 and 360 K for the normal mode analysis.

#### 2.2.2. Data processing

The data were processed with *CrysAlis PRO* (Rigaku OD, 2016 ▸) and the structures were refined with *SHELXL* (Sheldrick, 2015 ▸) in a sequential manner as described in Chernyshov *et al.* (2019 ▸) and Bogdanov *et al.* (2021 ▸). After inspecting the tem­per­ature-dependent ADPs of α-FOX-7 and removing the outliers, we decided to use 10 tem­per­ature points with 20–40 K steps, which adequately define the continuous smooth ADP curves in the range 80–360 K for the normal mode analysis. *OLEX2* (Dolomanov *et al.*, 2009 ▸; Bourhis *et al.*, 2015 ▸) was then used for the refinement with nonspherical scattering functions (Kleemiss *et al.*, 2021 ▸). Note that in the range 80–360 K, the diffraction θ_full_ and θ_max_ values of 23.49 and 32.72–32.97° yield respective data coverages of 89.3–90.9 and 66.0–67.3%. Although the data extended to sin θ/λ ≃ 0.8, the *I*/σ ratios in the outer shells were rather poor, in particular at *T* > 280 K (Table 1 ▸).

#### 2.2.3. Simulation of Γ-point α-FOX-7 unit cell

Periodic plane-wave density functional theory (DFT) simulations were performed in *CASTEP* (Version 20.11; Clark *et al.*, 2005 ▸). The electronic structure was expanded in plane waves to a kinetic energy cut-off of 1200 eV, with a charge–density cut-off of 35.49 Å^−1^. The exchange correlation functional of Perdew–Burke–Ernzerhof (PBE) (Perdew *et al.*, 1996 ▸) was used, alongside the semi-empirical dispersion correction of Tkatchenko–Scheffler (TS) (Tkatchenko & Scheffler, 2009 ▸). The electronic wavefunction was accepted following convergence < 10^−13^ eV and the residual forces converged < 10^−4^ eV per atom. *Γ*-point frequencies and eigenvectors were simulated through the linear response method (Refson *et al.*, 2006 ▸), without explicit consideration for LO–TO splitting. Dynamical matrices were subsequently calculated on a 3 × 3 × 3 Monkhorst–Pack grid (Monkhorst & Pack, 1976 ▸), and inter­polated onto a 9 × 9 × 9 fine grid, with which the ADPs were calculated as implemented within the *CASTEP* suite.

#### 2.2.4. DFT calculations of inter­nal vibrational frequencies

The atomic coordinates of α-FOX-7 at 80 K were employed to estimate inter­nal vibrational frequencies. The structure was initially optimized with the semi-empirical PM3 method and was then fully re-optimized using DFT calculations in the gas phase at the B3LYP/6-311+G(2d,p) level of theory with the program *GAUSSIAN09* (Frisch *et al.*, 2009 ▸). The energy minimization converged smoothly to a global minimum. After scaling, the harmonic vibrational frequencies in the range 57–3546 cm^−1^ agree with the literature data (see §3.3).

## 3. Results and discussion

### 3.1. Crystal structure of α-FOX-7

**Figure 1:**
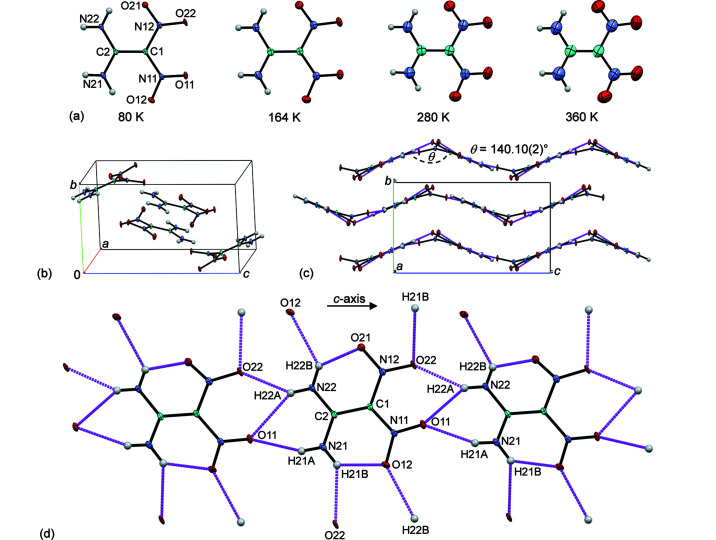
(*a*) Displacement ellipsoid plots of α-FOX-7 at 80, 164, 280 and 360 K (50% probability level). (*b*) Four mol­ecules in the monoclinic unit cell (*P*2_1_/*n*) of α-FOX-7 at 80 K. (*c*) The wave-shaped layer-type packing of α-FOX-7 at 80 K with the inter­planar angle of adjacent mol­ecules, θ = 140.10 (2)°, viewed along the *a* axis. (*d*) Intra- and inter­molecular O—H⋯O hydrogen bonds stabilizing the layer-type structure of α-FOX-7 at 80 K (magenta connecting lines). Note that atoms O21 and O22 having fewer inter­actions are oriented out of the mean mol­ecular plane.

**Figure 2:**
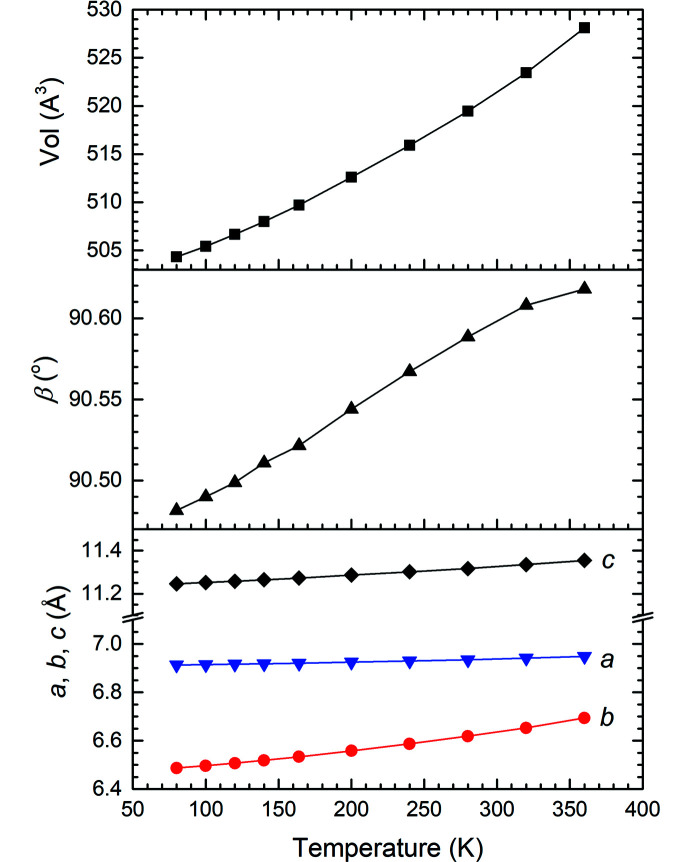
Unit-cell parameters of α-FOX-7 in the tem­per­ature range 80–360 K.

There are four α-FOX-7 mol­ecules in the monoclinic unit cell with the space group *P*2_1_/*n* [Fig. 1 ▸(*b*)]. The α-FOX-7 mol­ecule is nonplanar, as indicated by the larger deviations (Å) of atoms from the mean mol­ecular plane: O11 −0.209 (1), O21 −0.441 (1) and O22 0.761 (1), and the greater variations (∼9–37°) of the C2—C1—N11(N12)—O11(O21/O22) torsion angles from planarity for the 80 K data [Figs. 1 ▸(*a*) and 1 ▸(*b*)]. This is due to the steric hindrance between the two nitro groups and the small number of hydrogen-bonding inter­actions [Fig. 1 ▸(*d*)]. In the crystal, adjacent α-FOX-7 mol­ecules are closely connected *via* N—H⋯O hydrogen bonds along the *c* axis, forming herringbone layers with an obtuse inter­planar angle of 140.10 (2)° [Fig. 1 ▸(*c*)]. These layers are loosely packed along the *b* axis, allowing greater changes on this axis, as observed from the unit-cell volume expansion with increasing tem­per­ature (Fig. 2 ▸) and from the unit-cell volume contraction at high pressure (Hunter *et al.*, 2015 ▸).

### 3.2. Multi-tem­per­ature ADPs of α-FOX-7

**Figure 3:**
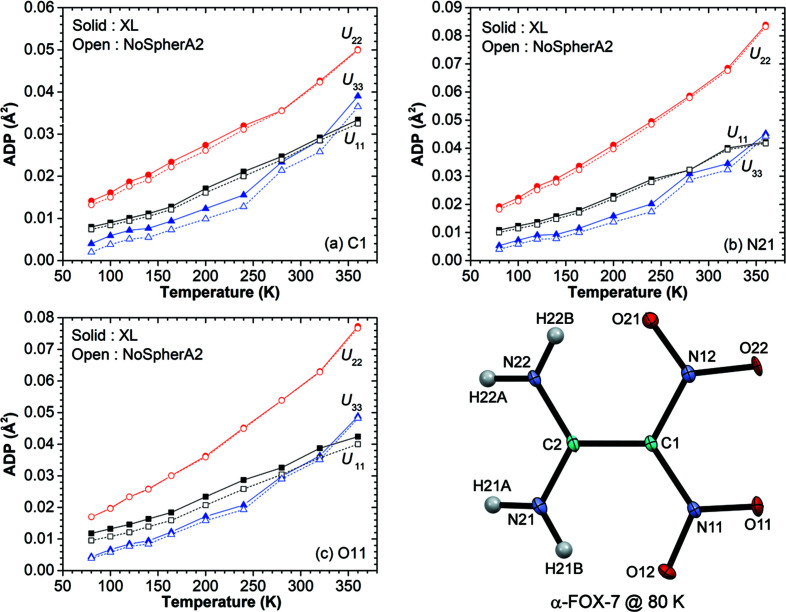
Multi-tem­per­ature ADPs of α-FOX-7 for atoms (*a*) C1, (*b*) N21 and (*c*) O11 from XL and NoSpherA2 refinements. The standard uncertainties are 3 × 10^−4^ Å^2^, or *ca* the line thickness. The displacement ellipsoid plot (50% probability level) with atom numbering is shown for α-FOX-7 at 80 K.

The multi-tem­per­ature ADPs of α-FOX-7 behave as expected within the harmonic approximation; see the principal elements *U*_11_, *U*_22_ and *U*_33_ for atoms C1, N21 and O11 [Figs. 3 ▸(*a*), 3 ▸(*b*) and 3 ▸(*c*), respectively]. In the classical regime, the ADPs increase linearly with tem­per­ature (80–164 K), but begin to increase more steeply at higher tem­per­atures, thereby indicating marked lattice anharmonicity in α-FOX-7. This anharmonicity is captured by the Grüneisen parameter (see §3.4). As the α-FOX-7 data do not cover the quantum regime (low tem­per­ature-independent limit), the theoretically tem­per­ature-independent ADPs, observed in the glycine polymorphs (Aree *et al.*, 2014 ▸), are not noticed here. Note that the elements *U*_11_ of all atoms increase more slowly at *T* > 300 K, resulting in an inter­section of the curves *U*_11_ and *U*_33_. This is probably due to the approach of the α-to-β phase transition at 389 K (Evers *et al.*, 2006 ▸), although the unit-cell parameters of α-FOX-7 do not show a discontinuity at the tem­per­ature of the present experiment (Fig. 2 ▸). The data resolution is not sufficient to really see the effects of bonding, so that the more elaborate charge–density description (NoSpherA2) does not have a large effect on the overall fit; see the ADP curves with open symbols in Figs. 3 ▸(*a*), 3 ▸(*b*) and 3 ▸(*c*). The *R*_1_ values are improved by 0.0046–0.0118 and the magnitudes of the highest peaks and deepest holes are decreased by 0.029–0.265 and 0.004–0.171 e Å^−3^, respectively (Table 1 ▸).

**Figure 4:**
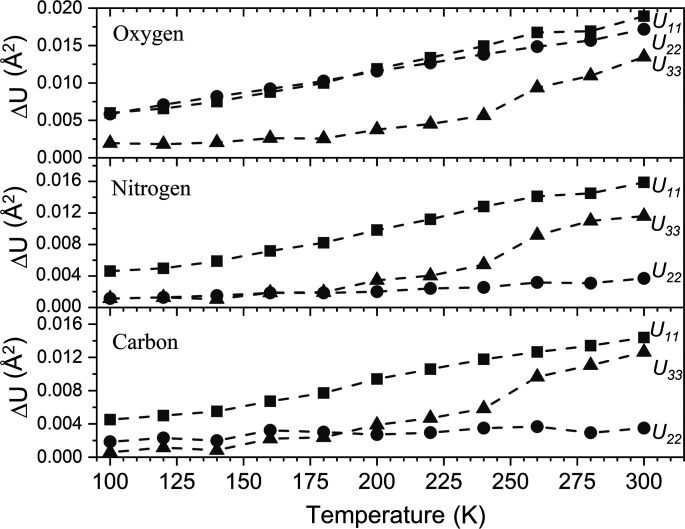
The average absolute deviations of ADPs for each atom type in α-FOX-7, with 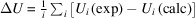 for the *i* atoms of each type. Values are shown as absolute deviations between the ADPs from the harmonic simulation and the diffraction experiment at each tem­per­ature.

We attempted to reproduce the variable-tem­per­ature ADPs of α-FOX-7 using periodic DFT simulations at the PBE-TS level of theory (Fig. 4 ▸). At the fully optimized geometry (*i.e.* the 0 K structure), our simulated harmonic ADPs consistently underestimate the magnitude of the primary displacement vectors, even at 100 K. This effect is, however, relatively small for both *U*_22_ and *U*_33_. This indicates a significant degree of anharmonicity in the FOX-7 structure, particularly in the direction between herringbone chains. Within the harmonic model, the mean-square atomic displacement increases approximately linearly with tem­per­ature. Thus, the growing deviation between our simulation from experiment with tem­per­ature is expected and consistent with the increased anharmonicity as the tem­per­ature rises. There is an intriguing divergence of *U*_33_ for all three atom types, occurring at *ca* 250 K. As observed in the experimental ADPs (Fig. 3 ▸), there is a rapid increase in motion along this direction at this tem­per­ature. As this direction corresponds to motion along the hydrogen-bonded herringbone chains, we can suggest that this increased divergence presumably reflects a weakening of the hydrogen-bonded chains with tem­per­ature. Further and dedicated efforts are ongoing to analyse this peculiar feature.

### 3.3. Inter­nal vibrations from DFT calculations

Upon scaling by a factor of 0.965, the 36 harmonic vibrational frequencies of α-FOX-7 obtained from the DFT/B3LYP/6-311+G(2d,p) calculation in the vacuum range from 57 to 3546 cm^−1^ agree overall with those from the MP2/6-31G(d,p) method (Sorescu *et al.*, 2001 ▸), the periodic DFT calculations using *CASTEP* (Averkiev *et al.*, 2014 ▸; Su *et al.*, 2019 ▸) and Raman spectroscopy (Dreger *et al.*, 2014 ▸) (Table 2 ▸). The three lowest frequencies (57, 92 and 115 cm^−1^) overlap with lattice frequencies and correspond to NO_2_ torsion, skeleton deformation and NH_2_ wagging modes, respectively. The 33 higher inter­nal vibration frequencies (203–3546 cm^−1^) were included for the calculation of the anisotropic tem­per­ature-independent contributions ɛ to the ADPs for H atoms, which were constrained in the normal mode analysis (Table 3 ▸). Note that the larger value of ɛ_33_ for H atoms is mainly attributed to the out-of-plane motions of higher frequencies (203–378 cm^−1^).

### 3.4. Crystal dynamics of α-FOX-7 from normal mode analysis

**Figure 5:**
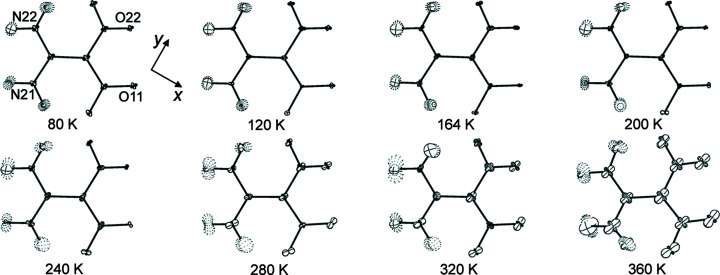
*PEANUT* plots showing the difference displacement parameters 3 × (*U*_obs_ – *U*_cal_) of α-FOX-7 from synchrotron diffraction (80–360 K); positive and negative differences are shown with respective solid and dashed lines. Axes shown are the mol­ecular coordinate system for normal mode analysis; see text for more details. The r.m.s. values of Σ(Δ*U*/σ_obs_) = ∼3–4 for non-H and ∼1 for H atoms.

We have two sets of α-FOX-7 ADPs deduced from spherical and nonspherical refinements with the respective programs *SHELXL * (XL) and *OLEX2* – NoSpherA2 (NoSph). There are three models of motions for parameterizing the variable-tem­per­ature ADPs. (i) Model *rbeg* stands for a typical rigid-body motion with three translations (*T_x_*, *T_y_* and *T_z_*) and three librations (*L_x_*, *L_y_* and *L_z_*), a Grüneisen constant for each of the six frequencies and two epsilons (the tem­per­ature-independent ADPs), each for the H and non-H atoms. (ii) Model *rbeg*+3*b* explicitly indicates the addition of three bending deformations of NO_2_ and CN_2_ groups (*U*_1_, *U*_2_ and *U*_3_) to *rbeg*. (iii) Model *rbeg*+3*b*+1*f* further includes one tem­per­ature-independent high frequency, which is attributed to CN_2_ wagging and NO_2_ twisting. The mol­ecular orientation is set as follows: the *x* axis passing through the N22→O11 vector is com­pleted with a right-hand rule by the *y* axis going through the N21→O22 vector (Fig. 5 ▸). The results of normal mode analysis are summarized in Table 3 ▸. The lattice vibrational frequencies from ADP analysis are com­pared to those derived from other techniques in Table 4 ▸. The model of motion *rbeg*+3*b*+1*f* provides estimated ADPs in fair agreement with the ADPs from diffraction, as depicted with the quite random distributions of difference displacement parameters (*U*_obs_ − *U*_cal_) in *PEANUT* plots (Hummel *et al.*, 1990 ▸) (Fig. 5 ▸).

Clearly, the *rbeg* model is insufficient to describe the large out-of-plane motions of the NO_2_ groups in α-FOX-7, as indicated by the rather high values of GOF > 5% and *wR*_2_ >> 10% for both sets of ADPs (Table 3 ▸). The model of motion is significantly improved by the addition of the deformations arising from bending, wagging and twisting of NO_2_ and CN_2_ groups, as evidenced from the values of GOF = 3.19 and *wR*_2_ = 9.30%. The six lattice vibrational frequencies (translations: 39.4, 44.6 and 56.6 cm^−1^; librations: 76.5, 85.2 and 97.5 cm^−1^) and one deformation frequency (145.5 cm^−1^) obtained are in line with those derived theoretically and spectroscopically, *i.e.* DFT-D and INS (Hunter *et al.*, 2015 ▸), and from Raman spectroscopy (Dreger *et al.*, 2014 ▸) (see Table 4 ▸). Moreover, the Grüneisen parameters (2.1–2.5) from ADP analysis are similar to those deduced from periodic Hartree–Fock calculations, *i.e.* 2.5 at 75 K and 1.0 at 300 K (Zerilli & Kuklja, 2007 ▸) and from tem­per­ature–pressure-variable synchrotron diffraction ex­peri­ments, *i.e.* 1.1 at ambient conditions (Zhang *et al.*, 2016 ▸). Note that if the six Grüneisen parameters were refined independently, the translational and librational frequencies ob­tained are mostly intact. The exception is the change of ∼15 cm^−1^, of which the libration 0.7*L_x_* – 0.7*L_z_* is com­pensated by the translation 0.6*T_x_* – 0.8*T_y_* + 0.1*T_z_*. The values of GOF = 2.50 and *wR*_2_ = 7.23% are improved, but the six Grüneisen parameters vary greatly, *i.e.* in the range 0.7–8.8 (Table 3 ▸). This suggests anharmonicity of the in-layer and out-of-layer vibrations due to the highly anisotropic thermal expansivities of α-FOX-7.

## 4. Conclusions

1,1-Di­amino-2,2-di­nitro­ethyl­ene, also known as DADNE or FOX-7, is an insensitive highly explosive material. Under ambient conditions, FOX-7 exists in an α-phase and it transforms to the β- and γ-forms at high-tem­per­ature–pressure due to the distinct 3D molecular arrangements, although the three phases are similarly constructed from herringbone layers of nonplanar mol­ecules due to the large out-of-plane deviations of the nitro O atoms. The low-frequency lattice vibrations, together with a com­plete set of X-ray diffraction data of α-FOX-7 covering a large tem­per­ature range (80–360 K), remain elusive. We therefore applied Bürgi’s method of concurrent analysis of multi-tem­per­ature atomic displacement parameters (ADPs) from diffraction data to explore the crystal dynamics of crystalline α-FOX-7.

Due to the abundance of inter­molecular N—H⋯O hydrogen bonds in α-FOX-7, the ADPs are minimally biased by the valence electron density. Hence, the ADPs derived from nonspherical refinement (NoSpherA2) with *OLEX2* (Dolo­manov *et al.*, 2009 ▸; Bourhis *et al.*, 2015 ▸) are slightly de­creased when com­pared to those from conventional spherical refinement with *SHELXL*. The variable-tem­per­ature non­spherical ADPs are suitably parameterized by a model of motion *rbeg*+3*b*+1*f*, which includes a typical rigid-body motion, a Grüneisen constant, two epsilons (the tem­per­ature-independent ADPs for H and non-H atoms), three bending deformations of NO_2_ and CN_2_ groups, and one tem­per­ature-independent high frequency (attributed to CN_2_ wagging and NO_2_ twisting). The anharmonicity arising from in-layer and out-of-layer vibrations is parameterized by the distinct Grüneisen parameters. In addition, we demonstrate that despite the limited quality of the diffraction data, the lattice vibrational frequencies from ADP analysis are reasonably close to those derived from inelastic scattering, Raman measurements and DFT calculations.

We conclude with a general note on experimentation. Single-crystal data collection with bright synchrotron radiation is fast, but com­plete and highly redundant ‘multi-run’ high-resolution data may allow sufficient time to see the effects associated with beam instabilities and radiation damage. Single-run data acquisitions, like those used here, rapidly map the tem­per­ature evolution of a crystal structure but suffer from reduced com­pleteness and redundancy. With the example of vibrational analysis based on tem­per­ature-dependent ADPs, we show that the useful and sometimes unique information content does not suffer from such a com­promise.

## Supplementary Material

Crystal structure: contains datablock(s) 080, 100, 120, 140, 164, 200, 240, 280, 320, 360, global. DOI: 10.1107/S2052520622002700/wu5002sup1.cif

Supporting information file. DOI: 10.1107/S2052520622002700/wu5002sup2.cml

CCDC references: 2163102, 2163103, 2163104, 2163105, 2163106, 2163107, 2163108, 2163109, 2163110, 2163111

## Figures and Tables

**Table 1:** Refinement statistics for α-FOX-7 from spherical (XL) and nonspherical (NoSpherA2) refinements

Temp(K)	No. of reflections [all, >2σ(I)]	*R*_1_(*F*)^*a*^ XL	*R*_1_(*F*)^*a*^ NoSpherA2	Δρ XL (e Å^−3^)		Δρ NoSpherA2 (e Å^−3^)	
80	1536, 1414	0.0364	0.0267	0.427	−0.400	0.256	−0.268
100	1536, 1400	0.0368	0.0261	0.481	−0.335	0.216	−0.206
120	1539, 1394	0.0381	0.0279	0.445	−0.340	0.213	−0.224
140	1540, 1389	0.0401	0.0299	0.426	−0.331	0.227	−0.222
164	1542, 1375	0.0400	0.0309	0.362	−0.339	0.228	−0.265
200	1560, 1363	0.0435	0.0334	0.343	−0.304	0.180	−0.186
240	1577, 1340	0.0502	0.0387	0.335	−0.386	0.207	−0.215
280	1576, 1222	0.0692	0.0574	0.616	−0.452	0.486	−0.318
320	1592, 1124	0.0849	0.0774	0.755	−0.472	0.709	−0.426
360	1607, 1029	0.0995	0.0949	0.710	−0.504	0.681	−0.501

Note: (*a*) *R*_1_(*F*) = Σ||*F*_o_| – |*F*_c_||/Σ|*F*_o_|.

**Table 2:** Comparison of inter­nal vibrational frequencies (cm^−1^) of α-FOX-7 from cal­cul­ations and Raman measurement

DFT^*a*^	MP2^*b*^	p-DFT^*c*^	p-DFT^*d*^	Exp.^*e*^	Assignment^*e*^
57	58	119	123		NO_2_ twist
92	108	151	194		NO_2_ twist
115	131	193	233		C—NH_2_ wag
203	155	270	253	246	NO_2_ rock, NH_2_ wag
280	276	317	316	318	NO_2_ rock, NH_2_ twist
299	311	324	331		NH_2_, NO_2_ rock
320	387	378	397	400	NH_2_ rock
369	395	441	448	457	NH_2_, NO_2_ rock
378	437	443	477	472	NH_2_ rock, NO_2_ twist, C—C st
432	459	469	490	481	NH_2_ twist, C—C st, NO_2_ sci
454	481	598	634		NH_2_ twist
454	491	610	646		NH_2_ wag
590	593	633	676	622	NH_2_ sci, twist
605	618	658	681		NH_2_ twist
666	625	668	695		NH_2_ wag
704	690	715	723		C—NO_2_ umb, NH_2_ twist
732	726	735	741	737	C—NO_2_ umb, NH_2_ twist
753	738	766	775	749	NH_2_ rock, NO_2_ sci
783	794	797	821	789	NH_2_ twist
841	862	833	843	856	NO_2_ sci, C—C st, NH_2_ rock
1041	1104	1006	1063	1024	NH_2_ rock
1049	1127	1050	1084	1070	NH_2_ rock
1106	1178	1106	1119	1142	NH_2_ rock, NO_2_ st (sym)
1172	1251	1141	1156	1165	NH_2_ rock, C–C st
1220	1378	1190	1201	1208	C—NO_2_ st (asym), NH_2_ sci
1287	1423	1300	1315	1311	C—NO_2_ st (sym), NH_2_ rock
1403	1538	1321	1339	1343	NH_2_ sci, NO_2_ st (asym)
1471	1588	1398	1411	1386	NH_2_ rock, C—C st, NO_2_ st (asym)
1505	1677	1480	1493	1506	NH_2_ sci, C—C st
1530	1714	1497	1519	1528	NH_2_ sci, C—C rock
1560	1752	1562	1599	1606	NH_2_ sci, C—C st
1581	1770	1603	1624	1630	NH_2_ sci, C—C rock
3341	3599	3288	3293	3299	NH_2_ st (sym)
3354	3609	3321	3334	3333	NH_2_ st (sym)
3544	3776	3418	3424	3405	NH_2_ st (asym)
3546	3776	3433	3450	3425	NH_2_ st (asym)

Notes: (*a*) DFT/B3LYP/6-311+G(2d,p) calculation in a vacuum; frequencies are scaled by a factor of 0.965 (this work). (*b*) MP2/6-31G(d,p) calculation in a vacuum; frequencies are scaled by a factor of 0.937 (Sorescu *et al.*, 2001 ▸). (*c*)/(*d*) Periodic-DFT (p-DFT) calculation (Averkiev *et al.*, 2014 ▸; Su *et al.*, 2019 ▸) using *CASTEP* (Clark *et al.*, 2005 ▸). (*e*) Raman measurement from solid sample with vibrational assignment: twist = twisting, wag = wagging, rock = rocking, scissor = scissoring, umb = umbrella, st = stretching, sym = symmetric and asym = asymmetric (Dreger *et al.*, 2014 ▸).

**Table 3:** Normal mode analysis of multi-tem­per­ature ADPs of α-FOX-7

	Frequency ν (cm^−1^) and eigenvector			Grüneisen	ɛ (× 10^−4^)^*a*,*b*^			GOF^*c*^	*wR*_2_ (%)^*d*^
	**ADP_NoSph; Model *rbeg*+3*b*+1*f***								
	*85.2 (48)*	*76.5 (46)*	*97.5 (29)*	2.5 (2)	Non-H-atoms			3.19	9.30
	*44.6 (6)*	*56.6 (11)*	*39.4 (5)*	2.5 (2)	−9 (3)	0 (1)	−3 (1)		
	*145.5 (32)*					−3 (3)	3 (1)	Obs: 840	
							25 (3)	Restr: 64	
*L_*x*_*	−0.607 (641)	0.702 (524)	−0.011 (105)					Param: 88	
*L_*y*_*	−0.317 (142)	0.054 (356)	0.306 (57)						
*L_*z*_*	−0.060 (120)	0.007 (75)	−0.947 (14)		H atoms				
*T_*x*_*	−0.739 (14)	−0.624 (14)	0.253 (22)		63	0	0		
*T_*y*_*	0.667 (14)	−0.731 (12)	0.143 (22)			174	0		
*T_*z*_*	0.096 (26)	0.274 (14)	0.957 (5)				369		
*U*_1_	−0.407 (565)	−0.602 (337)	0.034 (57)						
*U*_2_	0.492 (473)	0.375 (387)	0.041 (63)						
*U*_3_	0.345 (90)	0.042 (610)	0.080 (59)						
									
	**ADP_NoSph; Model *rbeg*+3*b***								
	*93.5 (26)*	*64.7 (27)*	*97.7 (58)*	2.4 (2)				3.16	9.19
	*44.5 (6)*	*56.2 (10)*	*39.5 (5)*	2.4 (2)				840/50/78	
									
	**ADP_NoSph; Model *rbeg*+3*b* [free 6 Grün.]**								
	*98.8 (41)*	*62.1 (13)*	*113.6 (64)*					2.50	7.23
	4.3 (8)	2.8 (4)	4.5 (10)					840/45/78	
	*44.1 (06)*	*71.0 (17)*	*38.3 (5)*						
	0.7 (4)	8.8 (3)	1.1 (3)						
									
	**ADP_NoSph; Model *rbeg***								
	*64.3 (16)*	*87.1 (33)*	*92.6 (51)*	2.1 (4)				6.24	18.4
	*45.2 (12)*	*57.6 (24)*	*38.0 (9)*	2.1 (4)				840/50/60	
									
	**ADP_XL; Model *rbeg***								
	*57.3 (12)*	*79.0 (24)*	*99.9 (61)*	2.3 (5)				5.06	15.2
	*43.9 (12)*	*55.0 (20)*	*42.2 (11)*	2.3 (5)				640/50/60	

Notes: (*a*) the H-atom epsilons are restrained to the values from DFT calculations. (*b*) The diagonal elements of epsilon for non-H atoms from DFT calculations are 13, 19, 13 × 10^−1^ Å^2^. (*c*) Goodness-of-fit (GOF) based on numbers of observations (Obs), restraints (Restr) and parameters (Param). (*d*) *wR*_2_ = [Σ*w*(*U*_obs_ − *U*_calc_)^2^/Σ*wU*_obs_^2^]^1/2^.

**Table 4:** Comparison of the lattice vibrational frequencies (cm^−1^) from ADP analysis, Γ-point simulation, DFT calculation, INS and Raman measurements

DFT-D^*a*^	INS^*a*^	Raman^*b*^	Γ-point^*e*^	ADP_Nosph^*f*^
29.3	27	25	35.7	39.4
48.1	46	46	48.6, 49.8	44.6
57.9	53	54	56.6, 57.1	56.6
60.1	58	63		
67.3	64	64	66.6	
76.3	69			
77.1	72	71	78.0	
81.5	79	77	81.4, 83.1	76.5
86.5	85		86.7, 87.4	85.2
91.7	88	90		
93.4	95	98	93.9	
97.3	97		97.8, 98.6	97.5
100.1	103	107	104.2	
109.3	112		111.5–118.4	
*119.6* ^*c*^	*117* ^*c*^			
*113.5* ^*c*^	*122* ^*c*^			
*122.9* ^*c*^	*138* ^*c*^			
124.4	145	139	125.8–139.2	
128.9	148	151	149.3	145.5
*130.9* ^*c*^	*150* ^*c*^	159	154.0, 156.8	
*145.4* ^*d*^	*162* ^*d*^	162	161.4	
*150.6* ^*d*^	*164* ^*d*^		167.4, 168.8	
*148* ^*d*^	*173* ^*d*^			
*165.7* ^*c*^	*177* ^*c*^			

Notes: (*a*) dispersion-corrected density functional theory (DFT-D) calculations and inelastic neutron scattering (INS) (Hunter *et al.*, 2015 ▸). (*b*) Raman spectroscopy under ambient conditions (room tem­per­ature and 1 atm) (Dreger *et al.*, 2014 ▸). (*c*) Deformational vibrations from CN_2_ wagging and NO_2_ twisting. (*d*) Deformational vibrations from NO_2_ twisting. (*e*) Simulated Γ-point of the α-FOX-7 unit cell (this work). (*f*) Normal mode analysis of nonspherical ADPs and model *rbeg*+3*b*+1*f* (this work).
